# *trans*-Bis[1,2-bis­(diphenyl­phosphan­yl)ethane]­chlorido(ethyn­yl)ruthenium(II)

**DOI:** 10.1107/S1600536812044558

**Published:** 2012-11-03

**Authors:** Alexander Trujillo, Mauricio Fuentealba, Ramiro Arratia-Perez, Judith A. K. Howard

**Affiliations:** a Departamento de Ciencias Quimicas, Facultad de Ciencias Exactas, Universidad Andres Bello, Avenida Republica 275, Santiago, Chile; b Instituto de Química, Facultad de Ciencias, Pontificia Universidad Católica de Valparaíso, Avenida Universidad 330, Curauma, Valparaíso, Chile; c Department of Chemistry, University of Durham, South Road, Durham DH1 3LE, England

## Abstract

The mol­ecular structure of the title compound, *trans*-[Cu(C_2_H)Cl(C_26_H_24_P_2_)_2_], consists of an Ru^II^ cation, located on an inversion centre, in an octa­hedral environment defined by two chelating phosphines, one acetyl­ide and one chloride ligand. The –C CH and the chlorine ligands are disordered over two equivalent positions (0.5 occupancy each). The coordination geometry is distorted octa­hedral, with the –C CH fragment and the Cl ligand in *trans* positions. The four P atoms occupy the equatorial plane of the octa­hedron and the chloride and acetyl­ide ligands the axial positions.

## Related literature

For details of electronic communication, see: Hu *et al.* (2005 ▶) and for mol­ecular electronics, see: Gauthier *et al.* (2008 ▶). For the chemistry of the *trans*-RuCl(C CH)(dppe)_2_, [dppe = 1,2-bis­(diphenyl­phosphan­yl)ethane] complex, see: Fox *et al.* (2009 ▶). For related structures, see: Faulkner *et al.* (1994 ▶); Zhu *et al.* (1999 ▶); Younus *et al.* (1999 ▶).
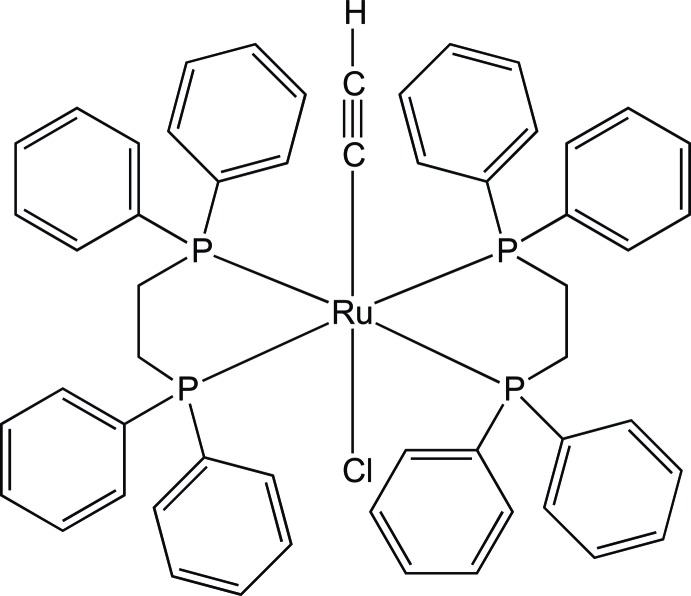


## Experimental

### 

#### Crystal data

[Cu(C_2_H)Cl(C_26_H_24_P_2_)_2_]
*M* *_r_* = 958.33
Monoclinic, 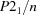

*a* = 10.92406 (18) Å
*b* = 16.0826 (2) Å
*c* = 13.2228 (2) Å
β = 105.2553 (17)°
*V* = 2241.22 (6) Å^3^
*Z* = 2
Mo *K*α radiation
μ = 0.59 mm^−1^
*T* = 120 K
0.41 × 0.35 × 0.20 mm

#### Data collection

Agilent Xcalibur (Sapphire3, Gemini ultra) diffractometer
Absorption correction: multi-scan (*CrysAlis PRO*; Agilent, 2011 ▶) *T*_min_ = 0.912, *T*_max_ = 1.000
32289 measured reflections
6470 independent reflections
5653 reflections with *I* > 2σ(*I*)
*R*_int_ = 0.040

#### Refinement

*R*[*F* ^2^ > 2σ(*F* ^2^)] = 0.030
*wR*(*F* ^2^) = 0.072
*S* = 1.09
6470 reflections
293 parameters
H-atom parameters constrained
Δρ_max_ = 0.49 e Å^−3^
Δρ_min_ = −0.48 e Å^−3^

### 

Data collection: *CrysAlis PRO* (Agilent, 2011 ▶); cell refinement: *CrysAlis PRO*; data reduction: *CrysAlis PRO*; program(s) used to solve structure: *SHELXS97* (Sheldrick, 2008 ▶); program(s) used to refine structure: *SHELXL97* (Sheldrick, 2008 ▶); molecular graphics: *OLEX2* (Dolomanov *et al.*, 2009 ▶).; software used to prepare material for publication: *OLEX2*.

## Supplementary Material

Crystal structure: contains datablock(s) global, I. DOI: 10.1107/S1600536812044558/bg2483sup1.cif

Structure factors: contains datablock(s) I. DOI: 10.1107/S1600536812044558/bg2483Isup2.hkl

Additional supplementary materials:  crystallographic information; 3D view; checkCIF report

## Figures and Tables

**Fig. 1.**
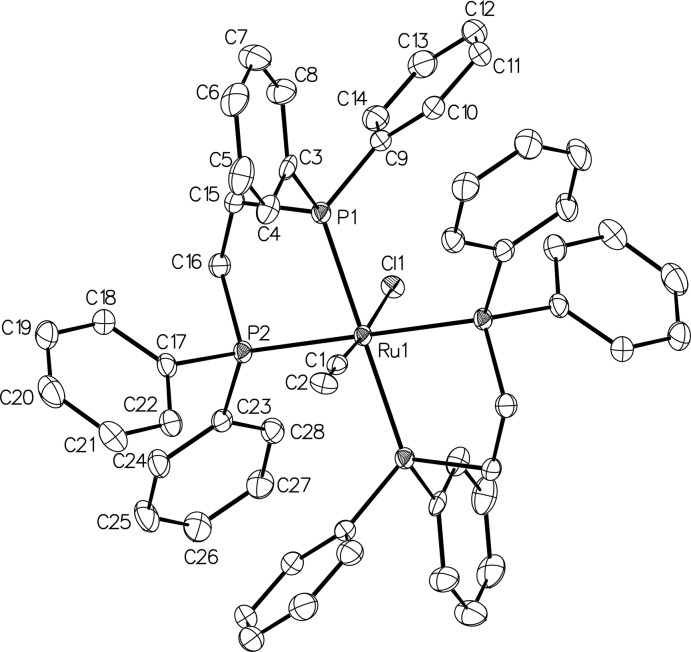
The molecular structure of title compound with full atom numbering scheme for indepent atoms. Displacement ellipsoids are presented at 50% probability level. Hydrogen atoms and disordered phenyl ring have been omitted in sake of clarity.

## supplementary crystallographic information

### Comment

In recent years, the design of carbon-rich organometallics compounds has been an interesting research topic, because in this type of structures the connection between the metal center with functional groups is achieved, and consequently the electronic communication is allowed through the C≡C unit (Hu *et al.*, 2005). Therefore, much attention has had the chemistry of the trans-RuCl(C≡CH)(dppe)2, [dppe= 1,2-Bis(diphenylphosphanyl)ethane] complex (Fox *et al.*, 2009). The Cl(dppe)2Ru—C≡C– endgroups behave as strongly electron-releasing groups which compares favorably to organic electron-releasing substituents such as methoxy or amino substituents. Moreover, in contrast to the organic substituents, these mononuclear organometallic acetylide complexes exhibit usually a very electron-rich chemistry and constitute a remarkable potential use in the molecular electronics field, since the oxidation state of the metal can be easily modulated (Gauthier *et al.*, 2008). In addition, these types of fragments have been shown to be interesting when they are attached to various unsaturated bridges. Depending on the nature of the bridge and the type of design obtained, they can exhibit different magnetic, optical or electronic properties (Faulkner *et al.*, 1994; Zhu *et al.*, 1999). Despite its important and widely use in the organometallic chemistry for the syntheses of vinylidene ruthenium complexes, for his rich electronic properties, the molecular structure of complex (1) has not been previously reported.

The main compound (1) crystallizes in the monoclinic space group *P*2_1_/*n*. The structure lies over a special position located in an inversion centre. the –C≡CH and the chlorine ligands are disordered over two equivalent positions (0.5 occupancy each one). The coordination geometry is a distorted octahedron with the –C≡CH fragment and the Cl ligand in *trans* position.

The structure of (1) shows four phosphorus atoms occupying the equatorial plane of the octahedron and the chloride and acetylide ligands occupying the axial position in *trans* configuration. The Cl—Ru, Ru—C(1), and C(1)—C(2) data for this complex fall within the range of those previously reported for related octahedral *trans*-bis(bidentate phosphine) ruthenium alkynyl complexes (Younus *et al.*, 1999).

Finally, both inter- and intramolecular hydrogen bonds or any other kind interaction are not observed in the crystalline packing of title compound.

### Experimental

A Schlenk tube under argon was loaded with [Ru(dppe)_2_Cl]^+^ (0.400 g, 0.369 mmol), ethynyltrimethylsilane (100 ml, 0.71 mmol), and 20 ml CH_2_Cl_2_ was added. The resulting mixture was stirred over night. After evaporation of the solvent, the residue was extracted with CH_2_Cl_2_ and chromatographied through a 2x4 cm Al_2_O_3_ column using CH_2_Cl_2_ as eluant. The solution was evaporated and the remaining solid was washed with *n-*pentane and dried *in vacuo*, to afford (1) as an yellow powder (0.262 g, 68,5%). Crystals were obtained by vapor diffusion of dichloromethane and hexane solution of (1).

### Refinement

The hydrogen atoms positions were calculated after each cycle of refinement with *SHELXL* (Sheldrick, 2008) using a riding model for each structure, with C—H distances in the range 0.95 to 0.98 Å. *U*_iso_(H) values were set equal to 1.2*U*_eq_. The ruthenium atom lies over an inversion center; in consequence, the dppe ligand occupy the equatorial position while chlorine and acetylide ligands are found on both axial positions. Both ligands were refined with 0.5 occupancies each one. On the other hand, phenyl ring (C3–C8) from dppe ligand is disordered in two positions with refined occupancies of 0.725 (17) and 0.275 (17). The anisotropic displacement parameters were constrained using the EADP instruction.

### Crystal data

**Table:**

[Cu(C_2_H)Cl(C_26_H_24_P_2_)_2_]	*F*(000) = 988
*M**_r_* = 958.33	*D*_x_ = 1.420 Mg m^−^^3^
Monoclinic, *P*2_1_/*n*	Mo *K*α radiation, λ = 0.7107 Å
*a* = 10.92406 (18) Å	Cell parameters from 13838 reflections
*b* = 16.0826 (2) Å	θ = 2.5–30.8°
*c* = 13.2228 (2) Å	µ = 0.59 mm^−^^1^
β = 105.2553 (17)°	*T* = 120 K
*V* = 2241.22 (6) Å^3^	Polyhedron, yellow
*Z* = 2	0.41 × 0.35 × 0.20 mm

### Data collection

**Table:**

Agilent Xcalibur (Sapphire3, Gemini ultra) diffractometer	6470 independent reflections
Radiation source: Enhance (Mo) X-ray Source	5653 reflections with *I* > 2σ(*I*)
Graphite monochromator	*R*_int_ = 0.040
Detector resolution: 16.1511 pixels mm^-1^	θ_max_ = 30.8°, θ_min_ = 2.5°
ω scans	*h* = −15→15
Absorption correction: multi-scan (*CrysAlis PRO*; Agilent, 2011)	*k* = −22→23
*T*_min_ = 0.912, *T*_max_ = 1.000	*l* = −18→18
32289 measured reflections	

### Refinement

**Table:**

Refinement on *F*^2^	Primary atom site location: structure-invariant direct methods
Least-squares matrix: full	Secondary atom site location: difference Fourier map
*R*[*F*^2^ > 2σ(*F*^2^)] = 0.030	Hydrogen site location: inferred from neighbouring sites
*wR*(*F*^2^) = 0.072	H-atom parameters constrained
*S* = 1.09	*w* = 1/[σ^2^(*F*_o_^2^) + (0.0263*P*)^2^ + 1.1519*P*] where *P* = (*F*_o_^2^ + 2*F*_c_^2^)/3
6470 reflections	(Δ/σ)_max_ < 0.001
293 parameters	Δρ_max_ = 0.49 e Å^−^^3^
0 restraints	Δρ_min_ = −0.48 e Å^−^^3^

### Special details

**Table:**

Experimental. Empirical absorption correction using spherical harmonics, implemented in SCALE3 ABSPACK scaling algorithm. (Agilent Technologies, 2011)
Geometry. All e.s.d.'s (except the e.s.d. in the dihedral angle between two l.s. planes) are estimated using the full covariance matrix. The cell e.s.d.'s are taken into account individually in the estimation of e.s.d.'s in distances, angles and torsion angles; correlations between e.s.d.'s in cell parameters are only used when they are defined by crystal symmetry. An approximate (isotropic) treatment of cell e.s.d.'s is used for estimating e.s.d.'s involving l.s. planes.
Refinement. Refinement of *F*^2^ against ALL reflections. The weighted *R*-factor *wR* and goodness of fit *S* are based on *F*^2^, conventional *R*-factors *R* are based on *F*, with *F* set to zero for negative *F*^2^. The threshold expression of *F*^2^ > σ(*F*^2^) is used only for calculating *R*-factors(gt) *etc*. and is not relevant to the choice of reflections for refinement. *R*-factors based on *F*^2^ are statistically about twice as large as those based on *F*, and *R*- factors based on ALL data will be even larger.

### Fractional atomic coordinates and isotropic or equivalent isotropic displacement parameters (Å^2^)

**Table:**

	*x*	*y*	*z*	*U*_iso_*/*U*_eq_	Occ. (<1)
Ru1	0.0000	0.0000	0.0000	0.01370 (5)	
Cl1	0.23554 (13)	0.03894 (6)	0.07756 (9)	0.0164 (2)	0.50
P1	−0.00132 (4)	0.01938 (2)	0.17791 (3)	0.01557 (8)	
P2	−0.03740 (4)	0.14451 (2)	−0.00604 (3)	0.01542 (8)	
C1	−0.1785 (5)	−0.0277 (2)	−0.0475 (3)	0.0159 (7)	0.50
C2	−0.2870 (5)	−0.0472 (3)	−0.0780 (4)	0.0182 (10)	0.50
H2	−0.3736	−0.0629	−0.1024	0.022*	0.50
C3	−0.1320 (5)	−0.0010 (3)	0.2384 (4)	0.0177 (7)	0.725 (17)
C4	−0.2548 (5)	−0.0110 (3)	0.1755 (4)	0.0240 (9)	0.725 (17)
H4	−0.2709	−0.0086	0.1013	0.029*	0.725 (17)
C5	−0.3543 (5)	−0.0244 (3)	0.2209 (6)	0.0328 (12)	0.725 (17)
H5	−0.4382	−0.0312	0.1779	0.039*	0.725 (17)
C6	−0.3308 (6)	−0.0280 (3)	0.3294 (6)	0.0332 (12)	0.725 (17)
H6	−0.3988	−0.0372	0.3605	0.040*	0.725 (17)
C7	−0.2080 (6)	−0.0180 (3)	0.3924 (5)	0.0354 (11)	0.725 (17)
H7	−0.1919	−0.0204	0.4665	0.042*	0.725 (17)
C8	−0.1085 (5)	−0.0046 (4)	0.3469 (4)	0.0276 (9)	0.725 (17)
H8	−0.0246	0.0022	0.3899	0.033*	0.725 (17)
C3A	−0.1411 (13)	−0.0092 (11)	0.2247 (11)	0.0177 (7)	0.275 (17)
C4A	−0.2629 (15)	0.0009 (12)	0.1584 (10)	0.0240 (9)	0.275 (17)
H4A	−0.2741	0.0161	0.0871	0.029*	0.275 (17)
C5A	−0.3695 (12)	−0.0117 (11)	0.1984 (12)	0.0328 (12)	0.275 (17)
H5A	−0.4522	−0.0067	0.1525	0.039*	0.275 (17)
C6A	−0.3573 (14)	−0.0301 (10)	0.2973 (13)	0.0332 (12)	0.275 (17)
H6A	−0.4299	−0.0460	0.3194	0.040*	0.275 (17)
C7A	−0.2455 (16)	−0.0268 (9)	0.3665 (11)	0.0354 (11)	0.275 (17)
H7A	−0.2396	−0.0303	0.4394	0.042*	0.275 (17)
C8A	−0.1349 (13)	−0.0181 (11)	0.3314 (11)	0.0276 (9)	0.275 (17)
H8A	−0.0544	−0.0181	0.3812	0.033*	0.275 (17)
C9	0.13110 (15)	−0.02627 (10)	0.27746 (12)	0.0187 (3)	
C10	0.12128 (17)	−0.10699 (11)	0.31425 (13)	0.0225 (3)	
H10	0.0450	−0.1376	0.2886	0.027*	
C11	0.22226 (19)	−0.14261 (12)	0.38806 (14)	0.0302 (4)	
H11	0.2148	−0.1974	0.4125	0.036*	
C12	0.33340 (19)	−0.09861 (14)	0.42603 (14)	0.0339 (5)	
H12	0.4022	−0.1230	0.4766	0.041*	
C13	0.34422 (18)	−0.01890 (13)	0.39024 (15)	0.0300 (4)	
H13	0.4207	0.0114	0.4163	0.036*	
C14	0.24357 (16)	0.01729 (12)	0.31609 (14)	0.0241 (4)	
H14	0.2519	0.0721	0.2918	0.029*	
C15	0.01206 (16)	0.13281 (10)	0.20793 (12)	0.0195 (3)	
H15A	−0.0705	0.1533	0.2157	0.023*	
H15B	0.0760	0.1413	0.2757	0.023*	
C16	0.04975 (16)	0.18398 (10)	0.12365 (12)	0.0190 (3)	
H16A	0.1423	0.1793	0.1321	0.023*	
H16B	0.0289	0.2433	0.1304	0.023*	
C17	−0.19892 (15)	0.18361 (10)	−0.02263 (12)	0.0180 (3)	
C18	−0.22999 (17)	0.24080 (11)	0.04617 (14)	0.0257 (4)	
H18	−0.1660	0.2599	0.1051	0.031*	
C19	−0.35332 (19)	0.27016 (13)	0.02957 (15)	0.0326 (4)	
H19	−0.3733	0.3085	0.0776	0.039*	
C20	−0.44647 (18)	0.24394 (13)	−0.05609 (16)	0.0329 (4)	
H20	−0.5309	0.2637	−0.0671	0.039*	
C21	−0.41672 (17)	0.18843 (12)	−0.12658 (15)	0.0289 (4)	
H21	−0.4806	0.1713	−0.1867	0.035*	
C22	−0.29422 (16)	0.15783 (10)	−0.10961 (13)	0.0225 (3)	
H22	−0.2751	0.1191	−0.1575	0.027*	
C23	0.02017 (15)	0.21231 (10)	−0.09510 (12)	0.0180 (3)	
C24	−0.05139 (18)	0.27775 (11)	−0.14948 (14)	0.0251 (4)	
H24	−0.1326	0.2895	−0.1395	0.030*	
C25	−0.0045 (2)	0.32590 (12)	−0.21821 (16)	0.0337 (4)	
H25	−0.0538	0.3706	−0.2547	0.040*	
C26	0.1133 (2)	0.30913 (12)	−0.23390 (16)	0.0318 (4)	
H26	0.1447	0.3419	−0.2814	0.038*	
C27	0.18501 (18)	0.24436 (11)	−0.18002 (14)	0.0265 (4)	
H27	0.2659	0.2326	−0.1908	0.032*	
C28	0.13984 (16)	0.19629 (10)	−0.11019 (13)	0.0214 (3)	
H28	0.1904	0.1525	−0.0727	0.026*	

### Atomic displacement parameters (Å^2^)

**Table:**

	*U*^11^	*U*^22^	*U*^33^	*U*^12^	*U*^13^	*U*^23^
Ru1	0.01802 (9)	0.00967 (8)	0.01496 (9)	0.00060 (6)	0.00706 (6)	0.00112 (6)
Cl1	0.0137 (7)	0.0166 (5)	0.0174 (5)	−0.0028 (5)	0.0015 (5)	0.0003 (3)
P1	0.01879 (19)	0.01286 (18)	0.01658 (19)	0.00130 (14)	0.00734 (15)	0.00160 (14)
P2	0.01964 (19)	0.01067 (17)	0.01706 (19)	0.00060 (14)	0.00677 (15)	0.00072 (14)
C1	0.022 (2)	0.0135 (16)	0.0144 (18)	−0.0002 (16)	0.0080 (15)	0.0004 (13)
C2	0.010 (2)	0.025 (2)	0.0165 (17)	−0.0044 (19)	−0.0013 (18)	0.0015 (13)
C3	0.0248 (11)	0.0106 (14)	0.0214 (16)	0.0001 (9)	0.0124 (12)	−0.0042 (12)
C4	0.0246 (12)	0.022 (2)	0.0288 (18)	0.0007 (12)	0.0125 (13)	−0.0040 (14)
C5	0.0248 (16)	0.027 (2)	0.051 (3)	−0.0008 (13)	0.0187 (18)	−0.0077 (18)
C6	0.035 (2)	0.0332 (12)	0.041 (3)	0.0019 (15)	0.027 (3)	0.0003 (19)
C7	0.026 (3)	0.0582 (19)	0.026 (2)	0.013 (2)	0.014 (2)	0.0114 (17)
C8	0.022 (2)	0.040 (2)	0.0226 (17)	0.0032 (15)	0.0101 (16)	0.0014 (13)
C3A	0.0248 (11)	0.0106 (14)	0.0214 (16)	0.0001 (9)	0.0124 (12)	−0.0042 (12)
C4A	0.0246 (12)	0.022 (2)	0.0288 (18)	0.0007 (12)	0.0125 (13)	−0.0040 (14)
C5A	0.0248 (16)	0.027 (2)	0.051 (3)	−0.0008 (13)	0.0187 (18)	−0.0077 (18)
C6A	0.035 (2)	0.0332 (12)	0.041 (3)	0.0019 (15)	0.027 (3)	0.0003 (19)
C7A	0.026 (3)	0.0582 (19)	0.026 (2)	0.013 (2)	0.014 (2)	0.0114 (17)
C8A	0.022 (2)	0.040 (2)	0.0226 (17)	0.0032 (15)	0.0101 (16)	0.0014 (13)
C9	0.0226 (8)	0.0195 (8)	0.0156 (7)	0.0052 (6)	0.0074 (6)	0.0015 (6)
C10	0.0300 (9)	0.0200 (8)	0.0189 (8)	0.0050 (7)	0.0087 (7)	0.0003 (6)
C11	0.0444 (11)	0.0243 (9)	0.0211 (9)	0.0152 (8)	0.0073 (8)	0.0041 (7)
C12	0.0346 (10)	0.0415 (12)	0.0221 (9)	0.0197 (9)	0.0014 (8)	−0.0002 (8)
C13	0.0219 (8)	0.0421 (11)	0.0254 (9)	0.0057 (8)	0.0050 (7)	−0.0037 (8)
C14	0.0233 (8)	0.0280 (9)	0.0228 (8)	0.0016 (7)	0.0091 (7)	0.0010 (7)
C15	0.0284 (8)	0.0133 (7)	0.0173 (7)	0.0024 (6)	0.0068 (6)	−0.0014 (6)
C16	0.0238 (8)	0.0130 (7)	0.0199 (7)	−0.0013 (6)	0.0054 (6)	−0.0006 (6)
C17	0.0217 (8)	0.0126 (7)	0.0214 (8)	0.0024 (6)	0.0086 (6)	0.0035 (6)
C18	0.0319 (9)	0.0235 (9)	0.0222 (8)	0.0087 (7)	0.0080 (7)	0.0011 (7)
C19	0.0386 (11)	0.0331 (10)	0.0310 (10)	0.0178 (8)	0.0177 (8)	0.0057 (8)
C20	0.0269 (9)	0.0345 (11)	0.0394 (11)	0.0123 (8)	0.0124 (8)	0.0128 (9)
C21	0.0246 (9)	0.0259 (9)	0.0333 (10)	0.0017 (7)	0.0023 (7)	0.0072 (7)
C22	0.0265 (8)	0.0154 (7)	0.0256 (8)	0.0007 (6)	0.0067 (7)	0.0019 (6)
C23	0.0253 (8)	0.0118 (7)	0.0184 (7)	−0.0018 (6)	0.0085 (6)	−0.0002 (6)
C24	0.0310 (9)	0.0179 (8)	0.0306 (9)	0.0060 (7)	0.0154 (7)	0.0067 (7)
C25	0.0456 (11)	0.0212 (9)	0.0405 (11)	0.0101 (8)	0.0222 (9)	0.0146 (8)
C26	0.0446 (11)	0.0222 (9)	0.0364 (10)	0.0009 (8)	0.0243 (9)	0.0081 (8)
C27	0.0287 (9)	0.0250 (9)	0.0303 (9)	−0.0010 (7)	0.0155 (7)	0.0016 (7)
C28	0.0250 (8)	0.0173 (8)	0.0231 (8)	0.0007 (6)	0.0083 (6)	0.0018 (6)

### Geometric parameters (Å, º)

**Table:**

Ru1—P2	2.3575 (4)	C18—H18	0.9500
Ru1—P2^i^	2.3575 (4)	C18—C19	1.389 (2)
Ru1—P1^i^	2.3769 (4)	C15—H15A	0.9900
Ru1—P1	2.3769 (4)	C15—H15B	0.9900
Ru1—Cl1^i^	2.5838 (14)	C10—H10	0.9500
Ru1—Cl1	2.5838 (14)	C10—C11	1.390 (2)
Ru1—C1	1.936 (5)	C24—H24	0.9500
Ru1—C1^i^	1.936 (5)	C24—C25	1.390 (2)
P2—C23	1.8332 (16)	C13—H13	0.9500
P2—C17	1.8311 (16)	C13—C12	1.382 (3)
P2—C16	1.8414 (16)	C11—H11	0.9500
P1—C3	1.840 (3)	C11—C12	1.381 (3)
P1—C9	1.8337 (16)	C21—H21	0.9500
P1—C15	1.8644 (16)	C21—C22	1.388 (2)
P1—C3A	1.850 (11)	C21—C20	1.390 (3)
Cl1—C1^i^	0.670 (4)	C27—H27	0.9500
Cl1—C2^i^	0.576 (4)	C27—C26	1.383 (3)
C3—C4	1.3900	C22—H22	0.9500
C3—C8	1.3900	C20—H20	0.9500
C4—H4	0.9500	C20—C19	1.375 (3)
C4—C5	1.3900	C12—H12	0.9500
C5—H5	0.9500	C19—H19	0.9500
C5—C6	1.3900	C25—H25	0.9500
C6—H6	0.9500	C25—C26	1.383 (3)
C6—C7	1.3900	C26—H26	0.9500
C7—H7	0.9500	C1—Cl1^i^	0.670 (4)
C7—C8	1.3900	C1—C2	1.190 (5)
C8—H8	0.9500	C2—Cl1^i^	0.576 (4)
C23—C28	1.397 (2)	C2—H2	0.9500
C23—C24	1.393 (2)	C3A—C4A	1.397 (12)
C17—C18	1.397 (2)	C3A—C8A	1.402 (11)
C17—C22	1.396 (2)	C4A—H4A	0.9500
C16—H16A	0.9900	C4A—C5A	1.415 (11)
C16—H16B	0.9900	C5A—H5A	0.9500
C16—C15	1.526 (2)	C5A—C6A	1.312 (17)
C9—C14	1.390 (2)	C6A—H6A	0.9500
C9—C10	1.400 (2)	C6A—C7A	1.320 (15)
C14—H14	0.9500	C7A—H7A	0.9500
C14—C13	1.393 (3)	C7A—C8A	1.410 (13)
C28—H28	0.9500	C8A—H8A	0.9500
C28—C27	1.391 (2)		
			
P2^i^—Ru1—P2	180.0	C14—C9—P1	121.07 (13)
P2^i^—Ru1—P1	98.156 (13)	C14—C9—C10	118.74 (16)
P2—Ru1—P1^i^	98.157 (13)	C10—C9—P1	120.17 (13)
P2^i^—Ru1—P1^i^	81.844 (13)	C9—C14—H14	119.8
P2—Ru1—P1	81.843 (13)	C9—C14—C13	120.38 (18)
P2—Ru1—Cl1^i^	94.58 (2)	C13—C14—H14	119.8
P2^i^—Ru1—Cl1^i^	85.42 (2)	C23—C28—H28	120.0
P2—Ru1—Cl1	85.42 (2)	C27—C28—C23	120.07 (16)
P2^i^—Ru1—Cl1	94.58 (2)	C27—C28—H28	120.0
P1^i^—Ru1—P1	179.999 (19)	C17—C18—H18	119.6
P1—Ru1—Cl1^i^	99.20 (3)	C19—C18—C17	120.84 (17)
P1^i^—Ru1—Cl1	99.20 (3)	C19—C18—H18	119.6
P1—Ru1—Cl1	80.80 (3)	P1—C15—H15A	108.9
P1^i^—Ru1—Cl1^i^	80.80 (3)	P1—C15—H15B	108.9
Cl1—Ru1—Cl1^i^	180.00 (4)	C16—C15—P1	113.15 (11)
C1^i^—Ru1—P2^i^	93.73 (11)	C16—C15—H15A	108.9
C1—Ru1—P2^i^	86.27 (11)	C16—C15—H15B	108.9
C1^i^—Ru1—P2	86.27 (11)	H15A—C15—H15B	107.8
C1—Ru1—P2	93.73 (11)	C9—C10—H10	119.8
C1—Ru1—P1^i^	85.22 (11)	C11—C10—C9	120.46 (17)
C1^i^—Ru1—P1	85.22 (11)	C11—C10—H10	119.8
C1—Ru1—P1	94.78 (11)	C23—C24—H24	119.8
C1^i^—Ru1—P1^i^	94.78 (11)	C25—C24—C23	120.36 (16)
C1^i^—Ru1—Cl1	4.43 (12)	C25—C24—H24	119.8
C1—Ru1—Cl1	175.57 (12)	C14—C13—H13	119.8
C1^i^—Ru1—Cl1^i^	175.57 (12)	C12—C13—C14	120.37 (18)
C1—Ru1—Cl1^i^	4.43 (12)	C12—C13—H13	119.8
C1^i^—Ru1—C1	180.0	C10—C11—H11	119.9
C23—P2—Ru1	121.50 (5)	C12—C11—C10	120.24 (18)
C23—P2—C16	102.27 (7)	C12—C11—H11	119.9
C17—P2—Ru1	119.70 (5)	C22—C21—H21	119.8
C17—P2—C23	101.62 (7)	C22—C21—C20	120.35 (18)
C17—P2—C16	103.83 (7)	C20—C21—H21	119.8
C16—P2—Ru1	105.41 (5)	C28—C27—H27	119.7
C3—P1—Ru1	127.91 (17)	C26—C27—C28	120.58 (17)
C3—P1—C15	96.22 (18)	C26—C27—H27	119.7
C9—P1—Ru1	116.67 (5)	C17—C22—H22	119.8
C9—P1—C3	99.91 (19)	C21—C22—C17	120.42 (16)
C9—P1—C15	103.66 (8)	C21—C22—H22	119.8
C9—P1—C3A	103.0 (5)	C21—C20—H20	120.1
C15—P1—Ru1	108.57 (5)	C19—C20—C21	119.78 (17)
C3A—P1—Ru1	121.2 (5)	C19—C20—H20	120.1
C3A—P1—C15	101.4 (6)	C13—C12—H12	120.1
C2^i^—Cl1—Ru1	158.0 (5)	C11—C12—C13	119.81 (17)
C2^i^—Cl1—C1^i^	145.2 (7)	C11—C12—H12	120.1
C4—C3—P1	119.8 (2)	C18—C19—H19	119.9
C4—C3—C8	120.0	C20—C19—C18	120.18 (18)
C8—C3—P1	120.2 (2)	C20—C19—H19	119.9
C3—C4—H4	120.0	C24—C25—H25	119.8
C5—C4—C3	120.0	C26—C25—C24	120.43 (17)
C5—C4—H4	120.0	C26—C25—H25	119.8
C4—C5—H5	120.0	C27—C26—H26	120.2
C4—C5—C6	120.0	C25—C26—C27	119.56 (17)
C6—C5—H5	120.0	C25—C26—H26	120.2
C5—C6—H6	120.0	C2—C1—Ru1	177.7 (4)
C7—C6—C5	120.0	Cl1^i^—C2—H2	161.3
C7—C6—H6	120.0	C1—C2—H2	180.0
C6—C7—H7	120.0	C4A—C3A—P1	119.6 (9)
C6—C7—C8	120.0	C4A—C3A—C8A	115.4 (8)
C8—C7—H7	120.0	C8A—C3A—P1	122.4 (10)
C3—C8—H8	120.0	C3A—C4A—H4A	120.3
C7—C8—C3	120.0	C3A—C4A—C5A	119.3 (10)
C7—C8—H8	120.0	C5A—C4A—H4A	120.3
C28—C23—P2	118.30 (12)	C4A—C5A—H5A	119.1
C24—C23—P2	122.70 (13)	C6A—C5A—C4A	121.8 (11)
C24—C23—C28	118.99 (15)	C6A—C5A—H5A	119.1
C18—C17—P2	122.66 (13)	C5A—C6A—H6A	119.5
C22—C17—P2	118.88 (12)	C5A—C6A—C7A	121.0 (9)
C22—C17—C18	118.41 (15)	C7A—C6A—H6A	119.5
P2—C16—H16A	109.9	C6A—C7A—H7A	120.3
P2—C16—H16B	109.9	C6A—C7A—C8A	119.5 (9)
H16A—C16—H16B	108.3	C8A—C7A—H7A	120.3
C15—C16—P2	108.75 (11)	C3A—C8A—C7A	121.5 (10)
C15—C16—H16A	109.9	C3A—C8A—H8A	119.2
C15—C16—H16B	109.9	C7A—C8A—H8A	119.2
			
Ru1—P2—C23—C28	−38.82 (15)	C4—C5—C6—C7	0.0
Ru1—P2—C23—C24	139.91 (13)	C5—C6—C7—C8	0.0
Ru1—P2—C17—C18	125.95 (13)	C6—C7—C8—C3	0.0
Ru1—P2—C17—C22	−56.55 (14)	C8—C3—C4—C5	0.0
Ru1—P2—C16—C15	−51.85 (11)	C23—P2—C17—C18	−97.05 (15)
Ru1—P1—C3—C4	−16.3 (4)	C23—P2—C17—C22	80.45 (14)
Ru1—P1—C3—C8	165.22 (18)	C23—P2—C16—C15	−179.76 (11)
Ru1—P1—C9—C14	87.24 (14)	C23—C28—C27—C26	1.0 (3)
Ru1—P1—C9—C10	−91.31 (13)	C23—C24—C25—C26	0.4 (3)
Ru1—P1—C15—C16	−12.75 (13)	C17—P2—C23—C28	−174.82 (13)
Ru1—P1—C3A—C4A	−34.9 (14)	C17—P2—C23—C24	3.91 (16)
Ru1—P1—C3A—C8A	163.9 (8)	C17—P2—C16—C15	74.82 (12)
P2—Ru1—P1—C3	99.6 (2)	C17—C18—C19—C20	−0.9 (3)
P2^i^—Ru1—P1—C3	−80.4 (2)	C16—P2—C23—C28	78.06 (14)
P2^i^—Ru1—P1—C9	49.05 (6)	C16—P2—C23—C24	−103.21 (15)
P2—Ru1—P1—C9	−130.95 (6)	C16—P2—C17—C18	8.85 (16)
P2—Ru1—P1—C15	−14.37 (6)	C16—P2—C17—C22	−173.64 (13)
P2^i^—Ru1—P1—C15	165.63 (6)	C9—P1—C3—C4	−151.8 (3)
P2^i^—Ru1—P1—C3A	−77.8 (7)	C9—P1—C3—C8	29.7 (3)
P2—Ru1—P1—C3A	102.2 (7)	C9—P1—C15—C16	111.93 (12)
P2^i^—Ru1—Cl1—C1^i^	79.0 (14)	C9—P1—C3A—C4A	−167.6 (11)
P2—Ru1—Cl1—C1^i^	−101.0 (14)	C9—P1—C3A—C8A	31.2 (11)
P2^i^—Ru1—Cl1—C2^i^	89.1 (15)	C9—C14—C13—C12	0.1 (3)
P2—Ru1—Cl1—C2^i^	−90.9 (15)	C9—C10—C11—C12	0.1 (3)
P2—Ru1—C1—Cl1^i^	101.3 (14)	C14—C9—C10—C11	0.1 (2)
P2^i^—Ru1—C1—Cl1^i^	−78.7 (14)	C14—C13—C12—C11	0.0 (3)
P2—C23—C28—C27	177.65 (13)	C28—C23—C24—C25	0.4 (3)
P2—C23—C24—C25	−178.28 (15)	C28—C27—C26—C25	−0.2 (3)
P2—C17—C18—C19	178.71 (14)	C18—C17—C22—C21	−0.2 (2)
P2—C17—C22—C21	−177.77 (13)	C15—P1—C3—C4	103.1 (3)
P2—C16—C15—P1	41.29 (14)	C15—P1—C3—C8	−75.4 (3)
P1^i^—Ru1—P2—C23	−30.89 (6)	C15—P1—C9—C14	−32.02 (15)
P1—Ru1—P2—C23	149.11 (6)	C15—P1—C9—C10	149.43 (13)
P1—Ru1—P2—C17	−82.46 (6)	C15—P1—C3A—C4A	85.3 (12)
P1^i^—Ru1—P2—C17	97.54 (6)	C15—P1—C3A—C8A	−75.9 (10)
P1^i^—Ru1—P2—C16	−146.19 (6)	C10—C9—C14—C13	−0.2 (2)
P1—Ru1—P2—C16	33.81 (6)	C10—C11—C12—C13	−0.2 (3)
P1^i^—Ru1—Cl1—C1^i^	−3.5 (14)	C24—C23—C28—C27	−1.1 (3)
P1—Ru1—Cl1—C1^i^	176.5 (14)	C24—C25—C26—C27	−0.5 (3)
P1^i^—Ru1—Cl1—C2^i^	6.6 (15)	C21—C20—C19—C18	−0.5 (3)
P1—Ru1—Cl1—C2^i^	−173.4 (15)	C22—C17—C18—C19	1.2 (3)
P1—Ru1—C1—Cl1^i^	−176.6 (14)	C22—C21—C20—C19	1.5 (3)
P1^i^—Ru1—C1—Cl1^i^	3.4 (14)	C20—C21—C22—C17	−1.2 (3)
P1—C3—C4—C5	−178.5 (4)	C1—Ru1—P2—C23	−116.59 (12)
P1—C3—C8—C7	178.5 (4)	C1^i^—Ru1—P2—C23	63.41 (12)
P1—C9—C14—C13	−178.75 (13)	C1—Ru1—P2—C17	11.84 (12)
P1—C9—C10—C11	178.64 (13)	C1^i^—Ru1—P2—C17	−168.16 (12)
P1—C3A—C4A—C5A	−172.6 (13)	C1^i^—Ru1—P2—C16	−51.89 (12)
P1—C3A—C8A—C7A	169.8 (14)	C1—Ru1—P2—C16	128.11 (12)
Cl1^i^—Ru1—P2—C23	−112.23 (7)	C1—Ru1—P1—C3	6.5 (3)
Cl1—Ru1—P2—C23	67.77 (7)	C1^i^—Ru1—P1—C3	−173.5 (3)
Cl1^i^—Ru1—P2—C17	16.20 (7)	C1—Ru1—P1—C9	135.96 (13)
Cl1—Ru1—P2—C17	−163.80 (7)	C1^i^—Ru1—P1—C9	−44.04 (13)
Cl1^i^—Ru1—P2—C16	132.47 (6)	C1—Ru1—P1—C15	−107.46 (12)
Cl1—Ru1—P2—C16	−47.53 (6)	C1^i^—Ru1—P1—C15	72.54 (12)
Cl1—Ru1—P1—C3	−173.7 (2)	C1^i^—Ru1—P1—C3A	−170.9 (7)
Cl1^i^—Ru1—P1—C3	6.3 (2)	C1—Ru1—P1—C3A	9.1 (7)
Cl1—Ru1—P1—C9	−44.31 (7)	C1^i^—Ru1—Cl1—C2^i^	10 (2)
Cl1^i^—Ru1—P1—C9	135.69 (7)	C3A—P1—C3—C4	−35 (6)
Cl1—Ru1—P1—C15	72.28 (6)	C3A—P1—C3—C8	147 (6)
Cl1^i^—Ru1—P1—C15	−107.72 (6)	C3A—P1—C9—C14	−137.4 (6)
Cl1—Ru1—P1—C3A	−171.1 (7)	C3A—P1—C9—C10	44.1 (6)
Cl1^i^—Ru1—P1—C3A	8.9 (7)	C3A—P1—C15—C16	−141.5 (5)
C3—P1—C9—C14	−131.0 (2)	C3A—C4A—C5A—C6A	2.2 (13)
C3—P1—C9—C10	50.5 (2)	C4A—C3A—C8A—C7A	7.8 (14)
C3—P1—C15—C16	−146.3 (2)	C4A—C5A—C6A—C7A	9.1 (17)
C3—P1—C3A—C4A	128 (7)	C5A—C6A—C7A—C8A	−11.4 (18)
C3—P1—C3A—C8A	−33 (5)	C6A—C7A—C8A—C3A	2.7 (15)
C3—C4—C5—C6	0.0	C8A—C3A—C4A—C5A	−10.1 (13)
C4—C3—C8—C7	0.0		

Symmetry code: (i) −*x*, −*y*, −*z*.

## References

[bb1] Agilent (2011). *CrysAlis PRO* Agilent Technologies UK Ltd, Yarnton, England.

[bb2] Dolomanov, O. V., Bourhis, L. J., Gildea, R. J., Howard, J. A. K. & Puschmann, H. (2009). *J. Appl. Cryst.* **42**, 339–341.

[bb3] Faulkner, C. W., Ingham, S. L., Khan, M. S., Lewis, J., Long, N. J. & Raithby, P. R. (1994). *J. Organomet. Chem.* **482**, 139–145.

[bb4] Fox, M. A., Harris, J. E., Heider, S., Pérez-Gregorio, V., Zakrzewska, M. E., Farmer, J. D., Yufit, D. S., Howard, J. A. K. & Low, P. J. (2009). *J. Organomet. Chem.* **694**, 2350–2358.

[bb5] Gauthier, N., Olivier, C., Rigaut, S., Touchard, D., Roisnel, T., Humphrey, M. G. & Paul, F. (2008). *Organometallics*, **27**, 1063–1072.

[bb6] Hu, Q. Y., Lu, W. X., Tang, H. D., Sung, H. H. Y., Wen, T. B., Williams, I. D., Wong, G. K. L., Lin, Z. & Jia, G. (2005). *Organometallics*, **24**, 3966–3973.

[bb7] Sheldrick, G. M. (2008). *Acta Cryst.* A**64**, 112–122. 10.1107/S0108767307043930 18156677

[bb8] Younus, M., Long, N. J., Raithby, P. R., Lewis, J., Page, N. A., White, A. J. P., Williams, D. J., Colbert, M. C. B., Hodge, A. J. & Khan, M. S. (1999). *J. Organomet. Chem.* **578**, 198–209.

[bb9] Zhu, Y., Millet, D. B., Wolf, M. O. & Rettig, S. J. (1999). *Organometallics*, **18**, 1930–1938.

